# Efficacy and Safety of CO_2_ Laser Therapy Combined with Collagen Cream in Managing Vulvo-Vaginal Atrophy: A Randomized, Controlled Study on Symptom Relief and Microbiome Modulation

**DOI:** 10.3390/medicina62020314

**Published:** 2026-02-03

**Authors:** Maurizio Filippini, Jessica Sozzi, Neila Maria de Góis Speck, Irene Fusco, Fernanda Kesserling Tso, Ernesta Dores, Miriam Farinelli

**Affiliations:** 1 Department of Obstetrics and Gynecology, Ospedale di Stato, 47893 Cailungo, San Marino; sozzi.jessica@hotmail.com (J.S.); miriam.farinelli@iss.sm (M.F.); 2 Departamento de Ginecologia, Núcleo de Prevenção em Doenças Ginecológicas, Escola Paulista de Medicina Unifesp, Sao Paulo 04023-062, Brazil; nezespeck@uol.com.br (N.M.d.G.S.); fernandaktso@gmail.com (F.K.T.); 3 Department of Clinical Research and Practice, El.En. Group, 50041 Calenzano, Italy; i.fusco@deka.it; 4 Department UOC Emergency, Emergency Medicine, San Carlo Hospital Trust of Potenza, 85100 Potenza, Italy; ernestadores3@gmail.com

**Keywords:** vulvo-vaginal atrophy (VVA), CO_2_ laser therapy, collagen-based cream, vaginal microbiome, postmenopausal women

## Abstract

*Background and Objectives*: Vulvo-vaginal atrophy (VVA), a prevalent condition among postmenopausal women, significantly impairs quality of life through symptoms like vaginal dryness, dyspareunia, and burning. Non-hormonal treatments, such as CO_2_ laser therapy, have shown promise in managing VVA symptoms with minimal side effects. The addition of adjunctive treatments may enhance efficacy and mitigate possible adverse effects. To evaluate the combined efficacy and safety of CO_2_ laser therapy and a collagen-based cream in treating VVA and to explore their potential impact on the vaginal microbiome. *Materials and Methods*: This was a single-center, randomized, interventional. Sixty postmenopausal women diagnosed with VVA were randomized into two groups: a control group receiving laser-only treatment and a treatment group receiving laser therapy with daily collagen-based cream application. Primary outcome measures included symptom improvement on the Visual Analog Scale (VAS) for VVA-associated symptoms. Secondary outcomes involved microbiome composition analysis. *Results*: Both groups showed significant symptom improvement, with the combination therapy group demonstrating superior reductions in burning, dyspareunia, and vaginal dryness (*p* < 0.05). Microbiome analysis revealed increased levels of beneficial species (*Lactobacillus iners* and *Lactobacillus crispatus*) and decreased pathogenic bacteria (*Gardnerella vaginalis* and *Atopobium vaginae*) in the treatment group, though these changes were not statistically significant. Mild side effects, such as burning and swelling in the first days following the treatment, were less frequent in the combination therapy group, likely due to the anti-inflammatory effects of the collagen-based cream. *Conclusions*: This study provides evidence supporting the use of CO_2_ laser therapy with collagen-based cream as an effective and well-tolerated treatment for VVA in postmenopausal women, achieving significant symptom relief. The combined therapy approach holds potential for enhanced efficacy and reduced side effects compared to laser-only treatment, offering a promising alternative for women ineligible for hormone-based therapies.

## 1. Introduction

Vulvo-vaginal atrophy (VVA), also known as genitourinary syndrome of menopause (GSM), is a common condition in postmenopausal women, characterized by the thinning and inflammation of the vaginal walls due to a significant decrease in estrogen levels [1]. VVA manifests as a reduction in epithelial thickness, pallor, reduction in vascularization, decreased elasticity, and loss of vaginal rugae [2]. This results in significant discomfort and affects the quality of life, with symptoms including vaginal dryness, dyspareunia, vaginal itching, pain, burning, and bleeding in the vulvo-vaginal area during sexual intercourse. Additionally, the condition can lead to urinary symptoms like incontinence, dysuria, urgency urination, and frequent urinary tract infections.

Maintaining vaginal health is essential for active and healthy aging in middle-aged and older women. Estrogen is crucial for maintaining a healthy vaginal ecosystem by promoting an intact vaginal epithelium and supporting a balanced microflora dominated by *lactobacilli* [3,4]. The vaginal epithelium relies on estrogen for its development and function, ensuring elasticity and lubrication. During menopause, the decline in estrogen disrupts this balance, resulting in vaginal atrophy. Serum estrogen levels typically range from 40 to 400 pg/mL in premenopausal women but drop to less than 20 pg/mL post-menopause, which is the primary cause of estrogen deficiency and subsequent atrophy [5,6].

Several studies, such as the EVES Study, have documented the high prevalence of VVA among postmenopausal women [7]. This study included 1226 postmenopausal women aged 45–75, with 75.3% showing clinically confirmed VVA. These studies highlight the need for improved management of VVA due to its significant impact on daily life and intimate relationships. Healthcare providers must proactively recognize VVA to preserve urogenital and sexual longevity through hormonal and non-hormonal strategies. Clinical diagnosis of VVA involves genital examination to identify objective signs and the use of subjective scales to assess symptoms, particularly vaginal dryness [2]. VVA may serve as an early marker of poor general health, similar to vasomotor symptoms, emphasizing the need for standardized care to enhance physical, emotional, and mental well-being in women.

VVA is a progressive condition that responds well to timely and sustained treatment. Current treatments for VVA often involve low-dose vaginal estrogen therapy, which helps restore the vaginal epithelium and beneficial *lactobacilli* [8]. However, long-term safety concerns and the need for alternative treatments have led to the exploration of other options.

CO_2_ laser therapy has proven to be an innovative treatment method to treat VVA [9]. Briefly, laser therapy works by delivering controlled, fractional CO_2_ laser energy to the vaginal tissues. This energy is dispersed through micro-tensions in the tissue, stimulating the body’s natural healing response. The process promotes collagen remodeling and neovascularization (formation of new blood vessels), increasing tissue elasticity and moisture retention [9]. The laser treatment also encourages re-epithelialization, leading to a thickened, more resilient mucosal lining. This enhanced tissue structure can reduce VVA symptoms such as dryness, burning, and pain during intercourse, providing an effective, non-hormonal option for managing VVA, especially in women for whom hormone therapy is unsuitable.

Among CO_2_ laser systems, the SmartXide2 device has been specifically designed for fractional vaginal treatments, delivering controlled energy to stimulate collagen remodeling and mucosal regeneration [10]. Previous studies have shown that such treatments can alleviate symptoms of VVA and improve tissue elasticity and hydration, with generally mild and transient side effects [11]. However, local inflammation or discomfort may occasionally occur following the procedure. To mitigate these effects and potentially enhance tissue recovery, topical agents with moisturizing and re-epithelializing properties have been proposed. One such option is PALINGEN cream, a collagen-based medical device containing hydrolyzed collagen and amino acids that support epithelial healing. Combining CO_2_ laser therapy with a topical collagen-based cream may therefore represent a strategy to improve symptomatic outcomes and reduce procedure-related discomfort. This study was designed to evaluate the efficacy and safety of this combined approach in postmenopausal women with VVA.

This study aims to evaluate the efficacy and safety of combining PALINGEN cream and the Monalisa touch laser in treating vaginal atrophy.

## 2. Materials and Methods

### 2.1. Study Design

This was an investigator-initiated, monocentric, experimental, randomized, controlled, interventional study designed to evaluate the efficacy and safety of combined treatment using the MonaLisa Touch system (SmartXide2 V2LR laser, DEKA, Florence, Italy) and PALINGEN cream (Praevenio Pharma, Aversa, Italy) in treating VVA in postmenopausal women. The study was conducted at the UOC of Obstetrics and Gynecology, Ospedale di Stato della Repubblica di San Marino, between June and October 2024. The study was approved by the Republic of San Marino ethics committee, protocol number 50/CERS/2024 of 20 May 2024. All patients signed an informed consent form prior to participation. All procedures adhere to ethical standards set by the committee responsible for human experimentation (both institutional and national), as well as the Helsinki Declaration of 1975, as revised in 2008.

This study was retrospectively registered on ClinicalTrials.gov, a publicly accessible clinical trial registry maintained by the U.S. National Library of Medicine. The clinical trial registration code is NCT07246616, and the final registration date is 17 November 2025. The study was registered retrospectively because, at the time of study initiation and enrollment of the first participant, registration in a public clinical trial registry was not required by the local regulatory or institutional framework for investigator-initiated, non-profit interventional medical device studies. The authors subsequently proceeded with trial registration to align with evolving international standards on clinical research transparency and to comply with the journal’s editorial policies prior to submission of this manuscript.

### 2.2. Study Population

The study targeted a population of adult postmenopausal women diagnosed with VVA. The study involved a thorough screening process to identify and enroll patients meeting the study criteria.

Inclusion criteria were as follows:

- Postmenopausal women with an absence of menstrual periods for at least 12 months.
- Diagnosed with vulvo-vaginal atrophy and exhibiting related symptoms such as dryness, dyspareunia (both introitus and deep), bleeding during intercourse, itching, or burning, whether spontaneous or induced by treatments like radio or chemotherapy.
- Age between 35 and 75 years.
- Patients are non-responsive or dissatisfied with previous topical estrogen therapy or those with contraindications to local and/or systemic estrogen use.
- A negative PAP test result performed within the last three years.

Exclusion criteria were as follows:

- Current pregnancy or breastfeeding.
- Presence of preneoplastic or neoplastic lesions of the cervix, vagina, or vulva.
- Active genital or urinary tract infections.
- Dermatological contraindications to laser treatment.
- Ongoing hormonal therapy (systemic or local).
- Neurological and/or psychiatric disorders.
- Chronic systemic diseases of autoimmune or metabolic nature.

### 2.3. Intervention

The study involves two groups: a control group (Laser only) receiving only the MonaLisa Touch SmartXide2 laser treatment, and an intervention group (Laser + PALINGEN) receiving both the MonaLisa Touch SmartXide2 laser treatment and PALINGEN cream. Participants were randomized in a 1:1 ratio to either the laser-only group (Control) or the laser + PALINGEN group using a computer-generated random sequence prepared by an independent statistician. Allocation was concealed through sequentially numbered, opaque, sealed envelopes, which were opened by the study nurse only after patient enrolment. No stratification factors were applied. Due to the visible nature of the intervention (cream application versus no cream), neither participants nor treating clinicians were blinded to group allocation. However, laboratory personnel conducting the microbiome analyses were blinded.

In the Control Group, patients received three treatments with the SMARTXIDE2 laser using the MonaLisa Touch™ procedure, at day 0, 30 and 90.

In the PALINGEN Group, patients received three treatments with the SMARTXIDE2 laser using the MonaLisa Touch™ procedure, at day 0, 30 and 90. Starting from the day of the first treatment, patients also applied one dose of PALINGEN cream (containing hydrolyzed collagen) to the vulvar vestibule and vaginal introitus daily throughout the 90 days laser treatment period. The cream was supplied in 30 g tubes, and patients were instructed on its proper application by medical staff.

### 2.4. Laser Treatment

Vaginal laser resurfacing was performed using a CO_2_ laser device, specifically the SmartXide2 V2LR system [11]. This device operates with a wavelength of 10,600 nm, utilizing a mixture of CO_2_, nitrogen, and helium as its active medium. The CO_2_ laser targets intra- and extracellular water, which constitutes approximately 77% of the vaginal mucosal tissue, enabling high-energy absorption with minimal tissue penetration. This selective absorption allows for the sealing of small nerve endings, reducing discomfort, while its hemostatic effect minimizes blood vessel edema.

The SmartXide2 device uses a specialized D-Pulse energy delivery mode. The D-Pulse initiates with a high-peak power phase for rapid and controlled superficial ablation of the epithelial layer of the atrophic mucosa. This phase is tailored to remove epithelial components with low water content effectively. The D-Pulse then transitions to a secondary phase with lower peak power and an extended emission time, facilitating deeper laser penetration to stimulate tissue remodeling within the mucosa.

To further refine the treatment, the Stack mode was utilized, allowing precise control over vaporization depth and thermal impact by delivering multiple successive pulses at a single point. The Stack mode can be set from one to five pulses per area, reducing potential side effects and enhancing tissue response.

During the procedure, the CO_2_ laser was configured with a micro-ablative zone (DOT) power of 40 W, a dwell time of 1000 µs, a DOT spacing of 1000 µm, and a Stack level of 2. The laser probe was inserted directly into the vagina without using a speculum, lubricants, or anesthetics, as patients reported only mild discomfort during probe insertion and no pain during the laser application itself; however, transient burning and swelling were reported after treatment sessions, as described in the Safety section. The entire treatment lasted only a few minutes. Following this, the laser was applied to the vaginal introitus and vulva using a fractioned scanner handpiece (Vulvar Probe, DEKA, Florence, Italy). For this application, the laser was adjusted to a DOT power of 25 W, maintaining a dwell time of 1000 µs, a DOT spacing of 1000 µm, and a Stack level of 1.

### 2.5. PALINGEN Cream

PALINGEN cream is a Class III medical device designed for topical application to support tissue repair processes. It is used as an adjunctive treatment for various dermatological and mucosal conditions, including wound healing, ulcerations, venous ulcers, pressure sores, minor burns, skin irritations, and irritations or infections of external genitalia. Additionally, PALINGEN is indicated for managing excoriations, abrasions, sunburn, diaper rash, and incontinence-associated skin maceration.

The formulation contains multiple bioactive and soothing ingredients with known moisturizing, anti-inflammatory, and re-epithelializing properties. Its primary components include hydrolyzed collagen, which promotes wound healing and tissue regeneration, and a complex of plant extracts and protective agents: *Equisetum arvense*, *Plantago lanceolata*, bisabolol, *Aloe barbadensis*, amino acids, vitamin E, *Eugenia caryophyllata*, and excipients.

### 2.6. Bacterial Characterization

Vaginal swab samples were collected in sterile tubes containing 1 mL of DNA-RNA Shield (Zymo Research, Irvine, CA, USA) and shipped to the Department of Chemistry, University of Parma, Italy, for analysis. DNA was extracted using the ZymoBIOMICS DNA Miniprep kit (Zymo Research) following the manufacturer’s protocol.

Partial 16S rRNA gene sequences (V3 region) were amplified using the primer pair Probio_Uni/Probio_Rev. Amplicons were processed according to the Illumina 16S Metagenomic Sequencing Library Preparation Protocol (Part #15044223 Rev. B), including adapter overhang addition. Operational taxonomic units (OTUs) were defined at 100% sequence homology using DADA2; OTUs represented by fewer than two sequences per sample were excluded. Diversity measures (α- and β-diversity, UniFrac analysis) were calculated accordingly.

Taxonomic classification was performed using QIIME2 against the SILVA reference database. For Lactobacillus-specific profiling, ITS PCR amplification and sequencing were conducted, and reads were classified using an updated Lactobacillus ITS reference dataset.

### 2.7. Clinical Investigation Endpoints

#### 2.7.1. Primary Endpoint

The primary endpoint of this study was the improvement in symptoms associated with VVA after 30 (Visit 1) and 90 days (Visit 2) of treatment, as assessed by the Visual Analog Scale (VAS). Improvements were measured by the difference in VAS scores before and after the treatment for each patient.

#### 2.7.2. Secondary Endpoints

1. Evaluation of the vaginal microbiome state before and after 30 and 90 days of treatment. This included the level of biodiversity calculated through the observed Operational taxonomic units (OTUs), the percentage abundance of the *Lactobacillus* genus, and the composition of species within the *Lactobacillus* genus. The relative amounts of pathogenic bacteria were also assessed.
2. Assessment of symptom improvement using VAS scores after 30 days and 90 days of treatment compared to baseline.
3. Monitoring and recording the frequency and severity of adverse events (e.g., mild bleeding, mild vulvo-vaginal redness) from the start of treatment to 30- and 90- days post-treatment.

### 2.8. Visual Analog Scale (VAS)

The Visual Analog Scale (VAS) is a widely used tool in clinical research for assessing the intensity of symptoms experienced by patients [12]. It is a simple and effective method for quantifying subjective experiences such as pain, discomfort, and other symptoms associated with various medical conditions. The VAS ranged from 0 to 10, where 0–3 indicates mild symptoms, 4–7 indicates moderate symptoms, and 8–10 indicates severe symptoms.

Patients were asked to indicate their perceived symptom intensity by placing a mark on the line corresponding to their experience. This method allows for a continuous scale of measurement, capturing subtle differences in symptom intensity that might not be detected with categorical scales. In this study, the VAS is used to assess various symptoms associated with VVA, including dyspareunia, vaginal dryness, pain at the vaginal introitus, itching, burning, and urgency.

### 2.9. Microbiota Evaluation

The evaluation of the microbiota was conducted by analyzing changes in bacterial composition before treatment and after 30 and 90 days of treatment. This assessment involved several key parameters. Firstly, the biodiversity level was calculated using the observed operational taxonomic units (OTUs) index, which represents the richness in bacterial groups and ranges from 0% to 100%. A higher value indicated a greater number of bacterial groups in the examined sample. The percentage abundance of the *Lactobacillus* genus was measured relative to the entire bacterial population constituting the vaginal microbiota. This helped determine the proportion of beneficial bacteria present. The species composition within the *Lactobacillus* genus has been analyzed. The dominance of specific species indicated different Community State Types (CSTs) of the vaginal microbiota. For instance, the dominance of *Lactobacillus crispatus* suggested a CST type 1, *Lactobacillus gasseri* suggested a CST type 2, *Lactobacillus iners* suggested a CST type 3, and *Lactobacillus jensenii* suggested a CST type 5. Lastly, the percentage abundance of pathogenic bacteria was measured relative to the entire bacterial population constituting the vaginal microbiota. This helped in understanding the presence and proportion of harmful bacteria.

To assess the vaginal microbiota, samples was collected with a swab at the beginning and end of the treatment cycle. A genetic analysis technology, Next Generation Sequencing (NGS), was used to read the gene sequences of the entire microbiotic community and categorize them according to the families present in the patients’ vaginas. This analysis utilized 16S rRNA gene microbial profiling, which allowed for a highly accurate assessment of the bacteria present in the vagina from a simple vaginal fluid sample. This methodology, unlike those commonly used for traditional sample analysis, enabled the precise identification of bacterial strains, determining whether they were in excess or deficit compared to pre-treatment levels.

### 2.10. Data Collection

The clinical and medical history of the patients was collected during the screening visit, with particular attention to the following details as documented in the Case Report Form (CFR):

- Approximate date of onset of gynecological discomfort or symptoms reported by the patient (within the four months preceding the visit).
- Concomitant therapies or treatments undertaken in the three months prior to the start of the study.
- Clinical information on the patient’s general health status, including past or current medical conditions and smoking habits.
- Pharmacotherapy, including chronic use of any medication (prescription and over-the-counter, including dietary supplements).
- History of allergies or idiosyncratic reactions to medications.

A complete physical examination and a comprehensive gynecological assessment were conducted before the start of treatment and after 30 and 90 days. This included measurements of height and weight, symptom collection, pelvic examination, breast examination, and cytological assessment.

The information of the study subjects has been kept confidential and managed in accordance with applicable laws and regulations. The data collection system for this study used built-in security features for all data transmissions in both directions, preventing unauthorized access to participant information. Access to the system is controlled by a sequence of identification codes and passwords assigned to users, available only to authorized personnel who have completed the necessary training. All information recorded on the CFRs are traceable to the original documents in the patient’s file.

The treatments proposed in the study have been already administered to patients as part of the center’s normal clinical practice. Therefore, there have been no additional risks for patients beyond those associated with routine clinical care.

### 2.11. Data Analysis

The sample size was calculated based on the primary objective of the study. To evaluate the efficacy of the combined treatment (PALINGEN Group) compared to laser treatment alone (Control Group) by comparing the mean differences in VAS scores between baseline and Visit 1 and 2, a sample size of 30 subjects per group was determined. This results in a total calculated sample size of 60 subjects. This sample size allowed for the estimation of a confidence interval for the difference between two means, assuming a normal distribution of the underlying data, with a width not exceeding 2.6 VAS points (95% Confidence Interval). This calculation assumed equal standard deviations between the two groups, set at 2.5. The sample size was specifically calculated to detect differences in symptom relief on the VAS and was not powered to evaluate changes in microbiome composition.

Changes in VAS scores over time were analyzed using repeated measures ANOVA to evaluate the significance of symptom improvement within and between the two groups. Paired *t*-tests were used to compare within-group changes, and independent *t*-tests were employed to compare differences between the control and PALINGEN groups. Chi-square tests were used to compare the incidence of adverse events between the two groups. Demographic data and baseline characteristics of the study population were summarized using descriptive statistics. Means and standard deviations were calculated for continuous variables, while frequencies and percentages were reported for categorical variables. Independent *t*-tests and chi-square tests were used to compare baseline characteristics between the two groups. Although mixed-effects models were initially planned, they were not applied due to the sample size and data structure. We acknowledge this as a methodological limitation, since mixed models may provide a more robust framework for handling repeated measures data. Safety analysis focused on the incidence and severity of adverse events. The data were summarized descriptively, and the differences between the groups were analyzed using chi-square tests to determine any significant differences in the occurrence of adverse events.

A *p*-value of less than 0.05 was considered statistically significant for all tests. The results were presented with corresponding 95% confidence intervals to provide a comprehensive understanding of the treatment effects and their clinical relevance.

## 3. Results

### 3.1. Patients

**Figure 1:**
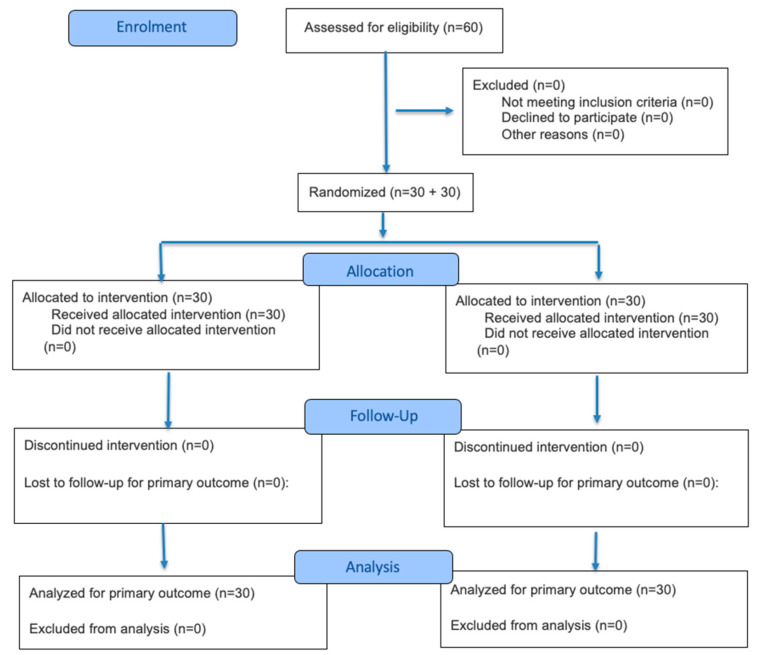
CONSORT 2025 flow diagram of participant progression through the trial. The diagram summarizes the number of participants assessed for eligibility, randomized, allocated to each intervention (laser-only vs. laser + PALINGEN cream), followed up, and included in the final analysis. All 60 enrolled participants met inclusion criteria, were randomized (30 per group), received the allocated intervention, completed follow-up, and were included in the primary and secondary outcome analyses.

A flow diagram of the progress through the phases of the randomized trial can be found in Figure 1, while the baseline characteristics and medical history of the study population are summarized in Table 1. A total of 60 unique patients were enrolled in the study, aged between 44 and 84 years, with a mean age of 57.12 (±7.63) years. All patients were postmenopausal and presented with varying degrees of VVA. Most patients experienced spontaneous menopause, with an average menopause age of 50.1 (±3.03 years). Each patient underwent three to four treatment sessions, with visits spaced approximately 30 days apart.

The baseline characteristics of the study population indicated that many patients had underlying comorbidities. Hypertension was the most prevalent condition, affecting 29.41% of the patients, followed by hypercholesterolemia in 20.59% of the patients. Osteoporosis was reported by 14.71%, while gastrointestinal disorders, such as reflux and gastritis, affected 11.76%. Thyroid disorders, primarily hypothyroidism, were present in 8.82% of the patients. Less common comorbidities included ischemic cerebral disease, which affected 5.88%, and psoriasis and asthma, each reported in 2.94% of the patients. The average age at menarche was 12.31 (±0.95) years. Regarding reproductive history, the average number of spontaneous deliveries was 1.69 (±0.87). Tumor history revealed that most of patients had no tumors, three patients had breast tumors, two had ovarian tumors, and two had thyroid tumors. Mood disorders were also evaluated, with six patients reporting insomnia and four experiencing mood changes. Stress was reported by six patients.

### 3.2. Efficacy

As expected, both the control (laser-only) and treatment (laser + PALINGEN) groups significantly reduced VVA symptoms. However, the treatment group consistently demonstrated greater efficacy in addressing infection-related symptoms, with statistically significant differences observed at specific time points. The relatively wide standard deviations in some VAS measures reflect variability in symptom perception among participants, which is common in studies using subjective patient-reported outcomes.

**Figure 2:**
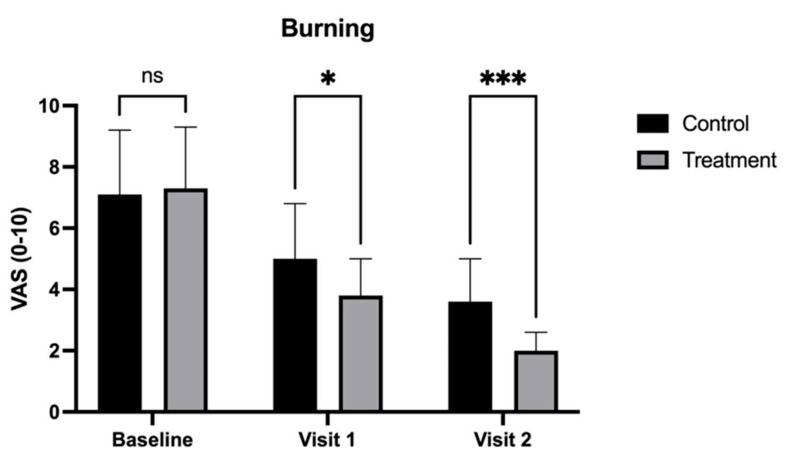
Visual Analog Scale (VAS) scores for burning sensation in patients treated with CO_2_ laser therapy alone (Control) and CO_2_ laser therapy combined with PALINGEN cream (Treatment) at baseline, Visit 1, and Visit 2. Bars represent mean VAS scores with standard deviation. At baseline, there was no significant difference (ns) between the control and treatment groups. By Visit 1, the treatment group showed a significantly greater reduction in burning sensation compared to the control group (* *p* < 0.05). By Visit 2, this difference was even more pronounced (*** *p* < 0.001), indicating enhanced symptom relief in the treatment group over time. Black bars represent the control group, and gray bars represent the treatment group.

**Figure 3:**
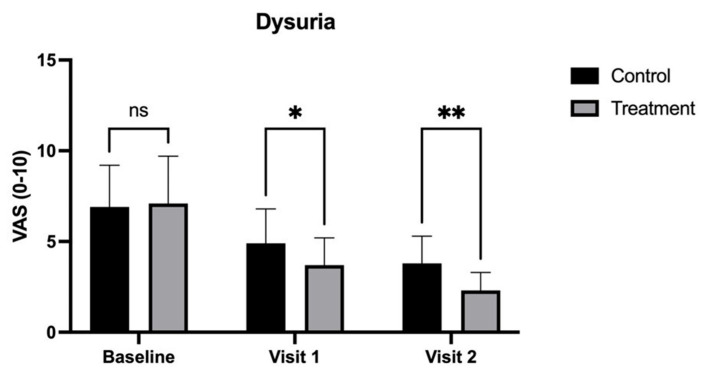
Visual Analog Scale (VAS) scores for dysuria in patients treated with CO_2_ laser therapy alone (Control) and CO_2_ laser therapy combined with PALINGEN cream (Treatment) at baseline, Visit 1, and Visit 2. Bars represent mean VAS scores with standard deviation. At baseline, there was no significant difference (ns) between the control and treatment groups. By Visit 1, the treatment group showed a significantly greater reduction in dysuria compared to the control group (* *p* < 0.05). By Visit 2, this difference became more pronounced (** *p* < 0.01), indicating enhanced symptom relief in the treatment group over time. Black bars represent the control group, and gray bars represent the treatment group.

At Visit 1 (30 days), burning and dysuria scores were significantly lower in the treatment group compared to the control group, indicating early enhanced relief. Specifically, burning scores were reduced to 3.8 (±1.2) in the treatment group versus 5.0 (±1.8) in the control group (*p* < 0.05) (Figure 2). Dysuria scores also showed a greater reduction in the treatment group, reaching 3.7 (±1.5) compared to 4.9 (±1.9) in the control group (*p* < 0.05) (Figure 3).

**Figure 4:**
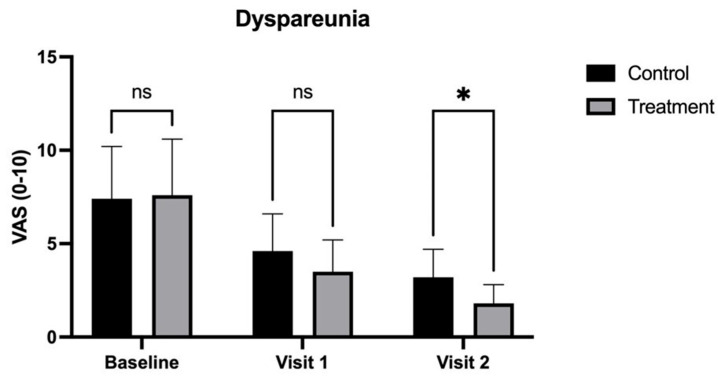
Visual Analog Scale (VAS) scores for dyspareunia in patients treated with CO_2_ laser therapy alone (Control) and CO_2_ laser therapy combined with PALINGEN cream (Treatment) at baseline, Visit 1, and Visit 2. Bars represent mean VAS scores with standard deviation. At baseline and Visit 1, there was no significant difference (ns) between the control and treatment groups. By Visit 2, however, the treatment group showed a significantly greater reduction in dyspareunia compared to the control group (* *p* < 0.05), indicating improved symptom relief over time with the combined therapy. Black bars represent the control group, and gray bars represent the treatment group.

**Figure 5:**
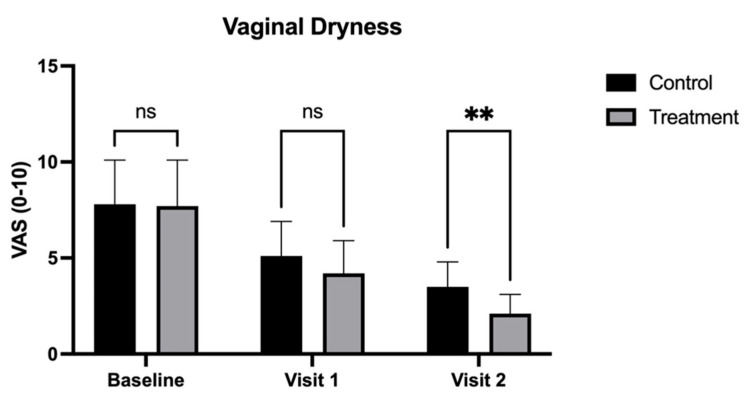
Visual Analog Scale (VAS) scores for vaginal dryness in patients treated with CO_2_ laser therapy alone (Control) and CO_2_ laser therapy combined with PALINGEN cream (Treatment) at baseline, Visit 1, and Visit 2. Bars represent mean VAS scores with standard deviation. At both baseline and Visit 1, there was no significant difference (ns) between the control and treatment groups. By Visit 2, however, the treatment group exhibited a significantly greater reduction in vaginal dryness compared to the control group (** *p* < 0.01), indicating improved effectiveness of the combined therapy over time. Black bars represent the control group, and gray bars represent the treatment group.

By Visit 2 (90 days), the treatment group demonstrated significantly greater improvements across most symptoms. Dyspareunia scores were significantly lower in the treatment group (1.8 ± 1.0) compared to the control group (3.2 ± 1.5, *p* < 0.05) (Figure 4). Vaginal dryness also showed a more pronounced reduction in the treatment group, with scores of 2.1 (±1.0) compared to 3.5 (±1.3) in the control group (*p* < 0.01) (Figure 5). Additionally, burning was notably lower in the treatment group (2.0 ± 0.6) than in the control group (3.6 ± 1.4, *p* < 0.001) (Figure 2), as was dysuria (treatment: 2.3 ± 1.0 vs. control: 3.8 ± 1.5, *p* < 0.01) (Figure 3). These results indicate that the treatment combination provided superior relief from these symptoms by the end of the study.

**Figure 6:**
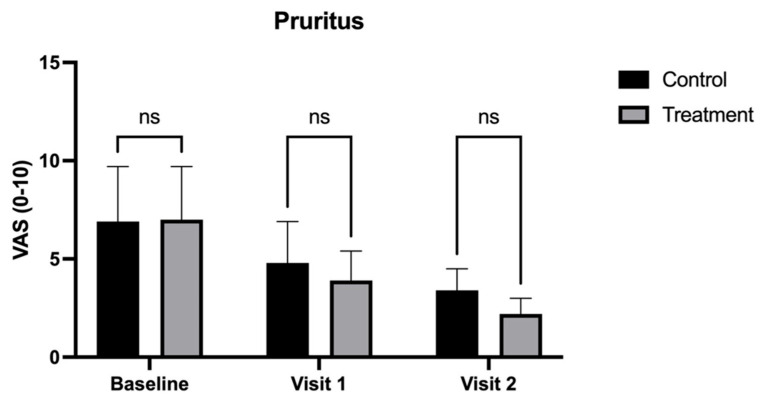
Visual Analog Scale (VAS) scores for pruritus in patients treated with CO_2_ laser therapy alone (Control) and CO_2_ laser therapy combined with PALINGEN cream (Treatment) at baseline, Visit 1, and Visit 2. Bars represent mean VAS scores with standard deviation. There was no significant difference (ns) between the control and treatment groups at baseline, Visit 1, or Visit 2, indicating that both treatments had a similar effect on pruritus reduction without a statistically significant advantage of the combined therapy. Black bars represent the control group, and gray bars represent the treatment group.

**Figure 7:**
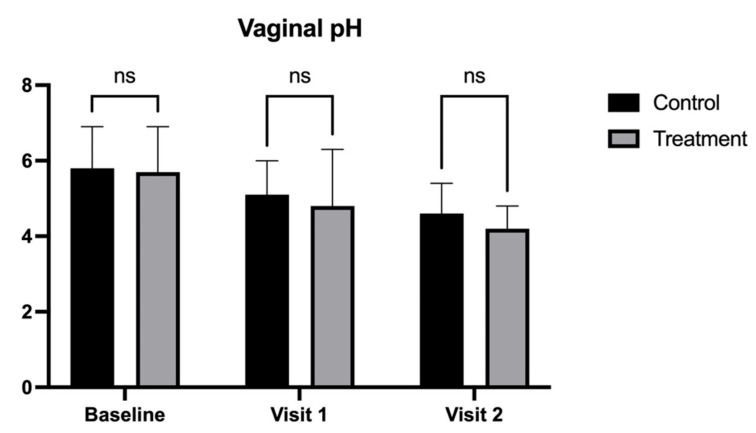
Vaginal pH measurement in patients treated with CO_2_ laser therapy alone (Control) and CO_2_ laser therapy combined with PALINGEN cream (Treatment) at baseline, Visit 1, and Visit 2. Bars represent mean pH scores with standard deviation. There was no significant difference (ns) between the control and treatment groups at baseline, Visit 1, or Visit 2, indicating that neither treatment had a statistically significant effect on vaginal pH. Black bars represent the control group, and gray bars represent the treatment group.

In contrast, pruritus (treatment: 2.2 ± 0.8 vs. control: 3.4 ± 1.1; Figure 6) and vaginal pH (treatment: 4.2 ± 0.6 vs. control: 4.6 ± 0.8; Figure 7) at Visit 2 (90 days), while lower in the treatment group, did not reach statistical significance compared to the control group (*p* < 0.05).

Overall, while both laser-only and laser + PALINGEN therapies effectively alleviated VVA symptoms, the addition of PALINGEN enhanced the treatment’s impact on key infection-related symptoms, demonstrating the efficacy of the combination therapy in managing VVA with infection-related discomfort.

### 3.3. Microbiome Analysis

**Figure 8:**
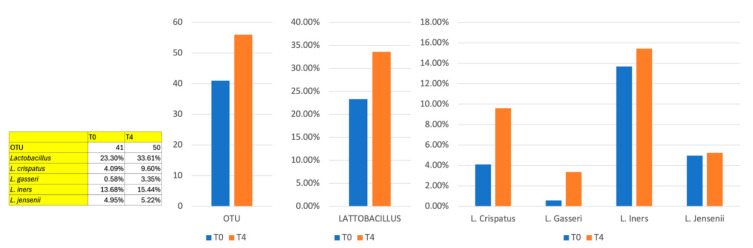
Lactobacilli strains analyzed after combined laser + Palingen cream treatment at baseline (T0, blue bars) and Visit 2 (T4, 90 days, orange bars).

The vaginal microbiome analysis conducted throughout the study revealed that neither the laser-only nor the laser + PALINGEN treatments produced statistically significant changes in any specific bacterial strain or genus. However, certain beneficial bacterial species, such as *Lactobacillus iners* and *Lactobacillus crispatus*, showed a trend to increase following treatment. *Lactobacillus iners* increased in prevalence during the treatment Visit 1 (30 days) and Visit 2 (90 days), while *Lactobacillus crispatus* was notably elevated at the final stages Visit 2 (90 days). Additionally, *Lactobacillus gasseri* increased in some patients, alongside a reduction in pathogenic bacteria (Figure 8).

**Figure 9:**
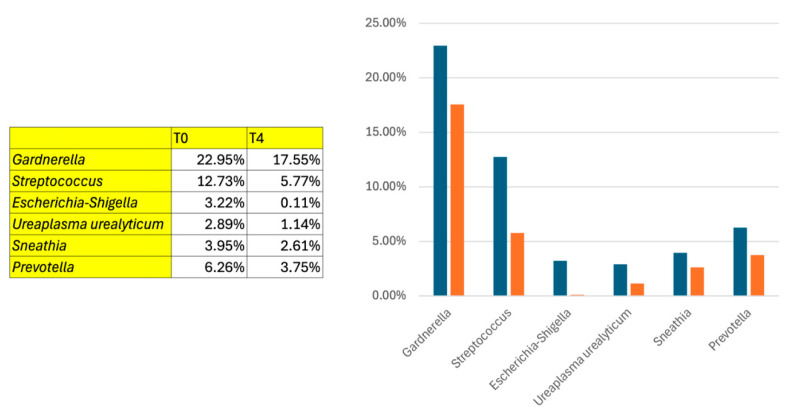
Pathogenic strains analyzed after combined laser + Palingen cream treatment at baseline (T0, blue bars) and Visit 2 (T4, 90 days, orange bars).

Conversely, several bacteria associated with adverse effects demonstrated reduced prevalence. *Gardnerella vaginalis,* a common infection-associated bacterium, showed a consistent decrease across treatment periods Visit 1 (30 days), and Visit 2 (90 days). Similarly, *Atopobium vaginae*, often linked with infections, decreased in some patients. Specific strains of *Prevotella* spp. also exhibited a reduced presence. Furthermore, *Ureaplasma urealyticum* showed a notable reduction primarily in the later stages Visit 2 (90 days) (Figure 9).

These observations indicate that, although no bacterial strain or genus was statistically modified by the treatments, the laser and laser + PALINGEN therapies contributed to a microbiome composition supportive of vaginal health by fostering beneficial species and reducing those associated with infections and inflammation. However, these microbiome observations should be considered exploratory, serving as preliminary trends rather than definitive outcomes. A summary of the main findings from microbiome analysis are reported in Table 2.

### 3.4. Safety

The safety profile of both the laser-only treatment and the combination of laser and PALINGEN cream was carefully monitored throughout the study (Table 3). Both treatments were generally well tolerated, with no serious adverse events reported in either group. Mild side effects were observed. These side effects were temporary and resolved without the need for additional intervention. The incidence of side effects was numerically higher but not statistically significant in the control group, where eight patients (26.7%) reported mild burning sensations, six (20.0%) redness, seven (23.3%) mild swelling, and three (10.0%) localized discomfort, compared to the treatment group, where five (16.7%) patients reported mild burning sensations, four (13.3%) redness, four (13.3%) mild swelling, and two (6.7%) localized discomfort. No patients discontinued treatment due to adverse events, and the reported side effects were all classified as mild, resolving within a few days post-treatment.

## 4. Discussion

This study assessed the efficacy, microbiome impact, and safety profile of laser-only therapy compared to combined laser therapy with PALINGEN cream in treating VVA symptoms in postmenopausal women. As expected, the findings indicate that both treatment modalities significantly alleviated symptoms associated with VVA. Nevertheless, our findings indicate that the combined therapy provided enhanced symptom relief, particularly in infection-related symptoms such as burning, dyspareunia, vaginal dryness, and dysuria. Additionally, although the treatments did not statistically alter specific bacterial strains or genera, the combined therapy appeared to foster beneficial changes in the vaginal microbiome, increasing beneficial species and decreasing pathogenic strains, which may support improved vaginal health.

Our primary findings align with prior research, such as studies on the efficacy of CO_2_ laser therapy in VVA, which documented significant symptom relief and improvements in vaginal health, particularly in patients unresponsive to traditional hormonal treatments [11,13,14]. For example, a previous study from our group found that fractional CO_2_ laser therapy with the SmartXide2 system effectively alleviated symptoms of vaginal atrophy, enhancing tissue elasticity and moisture levels, with minimal adverse effects [11]. Another study found that the fractional CO_2_ laser can not only be effective in treating vaginal atrophy and urinary symptoms, but also improves the quality of life and the sexual function of postmenopausal women [15].

Although effective, CO_2_ laser treatments have consistently been associated with side effects, although generally moderate and transient [9,11,13,15,16,17]. In our study, side effects were mitigated by the use of PALINGEN cream. The observed enhanced efficacy of laser therapy combined with PALINGEN cream may be attributed to PALINGEN’s re-epithelializing and moisturizing properties, which appear to support healing and reduce inflammation post-laser treatment. PALINGEN contains hydrolyzed collagen, which has shown beneficial effects in wound healing and inflammation reduction by promoting cellular re-epithelialization [18]. This may explain the greater reduction in symptoms like burning and dysuria observed in the combined treatment group, especially in early stages, as these symptoms are often exacerbated by inflammation and tissue damage.

In this study, we also analyzed the vaginal microbiome of the postmenopausal women enrolled. In terms of microbiome impact, there were no statistically significant changes in specific bacterial strains. Trends in data showed that beneficial strains such as *Lactobacillus iners* and *Lactobacillus crispatus* increased prevalence at later stages, particularly in the combined therapy group. These species are known for their protective roles in the vaginal environment, as they produce lactic acid, which maintains an acidic vaginal pH unfavorable to pathogenic bacteria [19]. Furthermore, a trend in reduction in species associated with vaginal infections, such as *Gardnerella vaginalis* and *Atopobium vaginae*, align with findings from studies suggesting that rebalancing the microbiome can reduce infection risk and support overall vaginal health [20,21].

It is important to underline that the microbiome results, while suggestive, require cautious interpretation. Rather than concluding definitive microbiome modulation, it is essential to acknowledge that observed changes could be incidental or influenced by factors outside the scope of this study. Future studies should include more robust statistical analyses to assess trends over extended time frames, allowing a more comprehensive understanding of how laser and combination therapies influence microbiome dynamics.

The safety profile of both treatments was favorable, with no serious adverse events reported. Mild, temporary side effects were slightly more prevalent in the laser-only group, with higher incidences of burning, redness, and swelling, likely due to inflammation associated with tissue remodeling post-laser treatment. The addition of PALINGEN cream appeared to mitigate these effects, consistent with its known anti-inflammatory properties, and no patient discontinued treatment due to adverse effects. This finding aligns with previous studies, demonstrating that collagen-based creams can enhance tissue healing and comfort following CO_2_ laser procedures [22,23]

This study has several limitations. Although randomization was conducted appropriately, the lack of blinding of participants and treating clinicians may have introduced subjective bias, particularly for patient-reported outcomes. The relatively small sample size, powered only for symptom relief and not for microbiome differences, also restricts the strength of conclusions. In addition, the single-center design may limit the generalizability of the findings, and the relatively short follow-up period prevents assessment of the long-term efficacy and safety of the combined therapy. Moreover, given the presence of multiple bioactive components, it is not possible to determine whether the observed effects of PALINGEN cream are attributable to hydrolyzed collagen alone or to synergistic actions of several ingredients. These aspects should be considered when interpreting the results, and future multicenter studies with larger cohorts and extended follow-up are warranted.

## 5. Conclusions

In conclusion, this study provides evidence that combining CO_2_ laser therapy with PALINGEN cream offers superior efficacy in managing VVA symptoms compared to laser treatment alone. The combination therapy shows potential for minimizing side effects, making it a promising approach for postmenopausal women with VVA, especially those who are unresponsive or contraindicated for hormonal treatments. Further research is warranted to confirm these findings and explore the long-term benefits of this combined approach in maintaining vaginal health.

## Figures and Tables

**Table 1:** Baseline characteristics and medical history of the study population.

Characteristic	Data
**Total Patients Enrolled**	60
**Age (years)**	Range: 44–84, Mean: 57.12 (±7.63)
**Postmenopausal Status**	100%
**Age at Menopause (years)**	Mean: 50.1 (±3.03)
**Treatment Sessions**	2 sessions (Visit 1 and Visit 2), spaced ~30 days apart
**Comorbidities**	
Hypertension	28.33% (*n* = 17)
Hypercholesterolemia	20.0% (*n* = 12)
Osteoporosis	15.0% (*n* = 9)
Gastrointestinal Disorders (reflux, gastritis)	11.67% (*n* = 7)
Thyroid Disorders (hypothyroidism)	8.33% (*n* = 5)
Ischemic Cerebral Disease	5.0% (*n* = 3)
Psoriasis	3.33% (*n* = 2)
Asthma	3.33% (*n* = 2)
**Age at Menarche (years)**	Mean: 12.31 (±0.95)
**Reproductive History**	
Spontaneous Deliveries (per woman)	Mean: 1.69 (±0.87)
**Tumor History**	
Breast Tumors	5.0% (*n* = 3)
Ovarian Tumors	3.33% (*n* = 2)
Thyroid Tumors	3.33% (*n* = 2)
**Mood Disorders**	
Insomnia	10.0% (*n* = 6)
Mood Changes	6.67% (*n* = 4)
Stress	10.0% (*n* = 6)

**Table 2:** This table summarizes the changes in vaginal bacterial species following laser and laser + PALINGEN treatments.

Bacterial Species	Observation	Time Points Noted
*Lactobacillus iners*	Increase in prevalence in later stages, enhancing protective flora	Visit 2 (90 days)
*Lactobacillus crispatus*	Significant increase primarily in final stages, stabilizing microbiome	Visit 2 (90 days)
*Lactobacillus gasseri*	Increase with concurrent reduction in pathogenic bacteria	Visit 1 (30 days), Visit 2 (90 days)
*Gardnerella vaginalis*	Consistent reduction, indicating decreased infection-associated bacteria	Visit 1 (30 days), Visit 2 (90 days)
*Atopobium vaginae*	Reduction in some patients, shifting away from infection-linked bacteria	Visit 1 (30 days), Visit 2 (90 days)
*Prevotella* spp.	Decreased presence of specific strains, aligning with reduced inflammation markers	Visit 1 (30 days), Visit 2 (90 days)
*Ureaplasma urealyticum*	Reduction primarily in later stages, indicating microbiome health improvement	Visit 2 (90 days)

**Table 3:** Observed side effects.

Side Effect	Control Group (*n* = 30)	Treatment Group (*n* = 30)
Mild Burning Sensation	8 (26.7%)	5 (16.7%)
Redness	6 (20.0%)	4 (13.3%)
Mild Swelling	7 (23.3%)	4 (13.3%)
Localized Discomfort	3 (10.0%)	2 (6.7%)

## Data Availability

The data that support the findings of this study are available from the corresponding author upon reasonable request.

## Abbreviations

The following abbreviations are used in this manuscript:

VVA	Vulvo-Vaginal Atrophy
GSM	Genitourinary Syndrome of Menopause
V2LR	Vulvo-Vaginal Laser Reshaping
